# Cost-Effectiveness Analysis of Five Competing Strategies for the Management of Multiple Recurrent Community-Onset *Clostridium difficile* Infection in France

**DOI:** 10.1371/journal.pone.0170258

**Published:** 2017-01-19

**Authors:** Emilie Baro, Tatiana Galperine, Fanette Denies, Damien Lannoy, Xavier Lenne, Pascal Odou, Benoit Guery, Benoit Dervaux

**Affiliations:** 1 Univ. Lille, CHU Lille, EA 2694 - Santé Publique: Epidémiologie et Qualité des Soins, Lille, France; 2 CHU Lille, Maladies Infectieuses, French Group of Faecal Microbiota Transplantation (GFTF), Lille, France; 3 CHU Lille, Direction de la Recherche en Santé, Lille, France; 4 Univ. Lille, CHU Lille, EA 7365 - GRITA - Groupe de Recherche sur les Formes Injectables et les Technologies Associées, Lille, France; 5 CHU Lille, Département d’Information Médicale, Lille, France; 6 Univ. Lille, CHU Lille, EA 7366 - Recherche Translationnelle: Relations Hôte-Pathogènes, Lille, France; University Hospital Llandough, UNITED KINGDOM

## Abstract

**Background:**

*Clostridium difficile* infection (CDI) is characterized by high rates of recurrence, resulting in substantial health care costs. The aim of this study was to analyze the cost-effectiveness of treatments for the management of second recurrence of community-onset CDI in France.

**Methods:**

We developed a decision-analytic simulation model to compare 5 treatments for the management of second recurrence of community-onset CDI: pulsed-tapered vancomycin, fidaxomicin, fecal microbiota transplantation (FMT) via colonoscopy, FMT via duodenal infusion, and FMT via enema. The model outcome was the incremental cost-effectiveness ratio (ICER), expressed as cost per quality-adjusted life year (QALY) among the 5 treatments. ICERs were interpreted using a willingness-to-pay threshold of €32,000/QALY. Uncertainty was evaluated through deterministic and probabilistic sensitivity analyses.

**Results:**

Three strategies were on the efficiency frontier: pulsed-tapered vancomycin, FMT via enema, and FMT via colonoscopy, in order of increasing effectiveness. FMT via duodenal infusion and fidaxomicin were dominated (i.e. less effective and costlier) by FMT via colonoscopy and FMT via enema. FMT via enema compared with pulsed-tapered vancomycin had an ICER of €18,092/QALY. The ICER for FMT via colonoscopy versus FMT via enema was €73,653/QALY. Probabilistic sensitivity analysis with 10,000 Monte Carlo simulations showed that FMT via enema was the most cost-effective strategy in 58% of simulations and FMT via colonoscopy was favored in 19% at a willingness-to-pay threshold of €32,000/QALY.

**Conclusions:**

FMT via enema is the most cost-effective initial strategy for the management of second recurrence of community-onset CDI at a willingness-to-pay threshold of €32,000/QALY.

## Introduction

*Clostridium difficile* infection (CDI) is the leading cause of healthcare associated diarrhea, presenting a significant burden to global healthcare systems [1]. In recent years, there has been an increase of incidence and severity of CDI in North America and Europe. Rates of community-acquired CDI have also increased and community-associated CDI is estimated to be responsible for more than one third of all CDI cases [2,3]. The main problem in CDI is symptomatic relapse after antimicrobial therapy completion. Moreover, the risk of recurrent CDI is increased in patients who have already had one recurrence, rising from 25% after an initial episode to 45% after a first recurrence and to 65% after two recurrences [4]. Recurrent CDI is associated with a diminished quality of life and increased morbidity. In addition, recurrent CDI also increases the risk of person-to-person transmission [4]. A recent study focusing on the economic consequences of recurrent CDI compared to patients with CDI who did not experience a recurrence showed that there were substantially higher pharmacological and hospitalization costs among the patients with recurrent CDI [5].

Treatment of multiple recurrent CDI remains challenging. In 2014, the European Society of Clinical Microbiology and Infectious Diseases (ESCMID) provided an overview of currently available CDI treatments [6]. Vancomycin use is recommended for treatment of multiple recurrent CDI, administered as a pulsed or tapered regimen. Fidaxomicin is also recommended for the treatment of multiple recurrent CDI. Nevertheless, both of these drugs are moderately supported by the ESCMID guideline (grade B-II). Fecal microbiota transplantation (FMT) consists of transplanting a fecal suspension from a healthy donor into a patient’s gastrointestinal tract through duodenal infusion, enema, or colonoscopy. FMT has been a successful therapeutic approach to recurrent CDI in numerous case series and in two randomized clinical trials [7,8]. The ESCMID endorses FMT as first-line therapy for patients who have had three or more CDI episodes with a strong recommendation (grade A-1).

Economic analyses compare different treatments in terms of clinical outcomes and costs [9]. To date, published cost-effectiveness analyses of CDI involving FMT have been performed in the USA and in Canada using cost data that may not apply to European countries [10–15]. The aim of this study was to analyze the cost-effectiveness of 5 strategies constructed from the ESCMID guideline for the management of multiple recurrence of CDI in adults where the first-line treatments were pulsed-tapered vancomycin, fidaxomicin, FMT via colonoscopy, FMT via duodenal infusion, and FMT via enema.

## Methods

### Model structure

**Fig 1:**
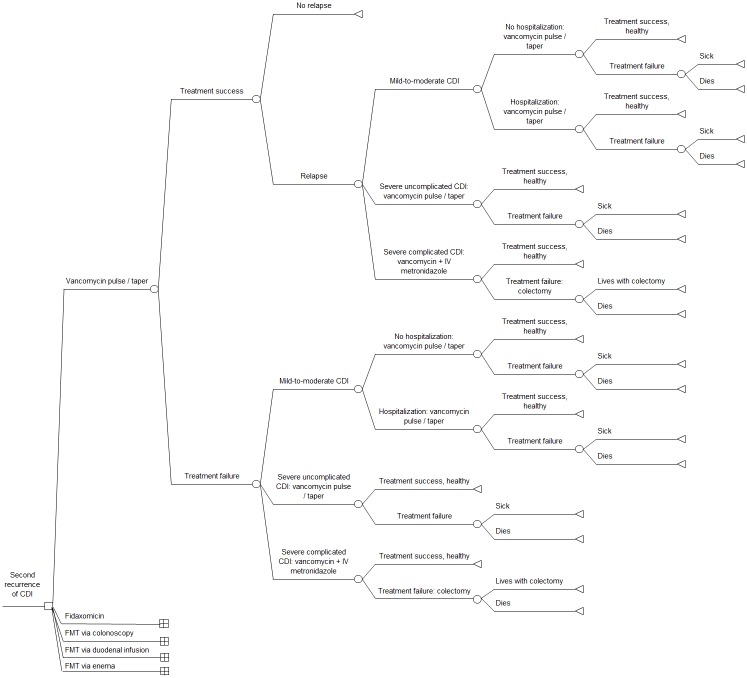
Decision tree comparing 5 strategies for the treatment of second recurrence of community-onset *Clostridium difficile* infection. Note: expanded model details shown for vancomycin pulse/taper arm only. Abbreviations: CDI: *Clostridium difficile* infection; FMT: fecal microbiota transplantation.

We conducted a decision-analytic model comparing 5 strategies for the management of second recurrence of CDI according to the ESCMID guideline (Fig 1). All analyses were performed using the TreePlan add-in (Decision Toolworks, San Francisco, USA) for Excel 2010 (Microsoft Corporation, USA) and PopTools [16]. Our model was based, in part, on previously published decision analytic models that investigated the potential value of FMT for CDI and cost-effectiveness analyses evaluating fidaxomicin versus vancomycin in CDI [10,12–15,17–20].

As defined in European guidelines, we considered recurrent CDI to be an episode occurring within 8 weeks after the onset of a previous episode that resolved after completion of the initial treatment [6]. Multiple recurrences were defined as more than two recurrences (i.e. more than 3 episodes). Treatment failure was defined as a need for further CDI therapy.

The patient modeled in the study was an adult experiencing a second recurrence (i.e. third occurrence) of mild-to-moderate CDI diagnosed at an outpatient visit (Fig A-E in S1 File). Following European guidelines, the first-line therapies for the strategies were pulsed-tapered vancomycin, fidaxomicin, FMT via colonoscopy, FMT via duodenal infusion, and FMT via enema [6]. Following treatment, patients were considered cured or treatment failures. Patients who failed treatment either had mild-to-moderate CDI, severe uncomplicated CDI, or severe complicated CDI. It was assumed that one medical consultation was required for each patient with CDI and that severe CDI required hospitalization. Patients initially cured could develop a recurrence of mild-to-moderate CDI, severe uncomplicated CDI, or severe complicated CDI. On the basis of published data, we assumed that recurrence appeared 10 days after treatment by pulsed-tapered vancomycin, 31 days after treatment by fidaxomicin, and 32 days after treatment by FMT [7,8,21–26]. To evaluate each treatment separately, we assumed that patients who failed therapy and patients who had a relapse were treated with the same treatment as their previous episode if they had mild-to-moderate CDI or severe uncomplicated CDI. Patients with severe complicated CDI received oral vancomycin plus intravenous metronidazole, as recommended in European guidelines [6]. Following treatment, they could be either cured or treatment failures requiring colectomy. We considered the possibility of adverse events and death from FMT via colonoscopy and FMT via duodenal infusion. Adverse events and death from FMT via colonoscopy were assumed to be equivalent to those of a colonoscopy procedure [27–30]. Similarly, due to a lack of current data, adverse events and death from FMT via duodenal infusion were assumed to be equivalent to adverse events and death from upper gastrointestinal endoscopy [31]. We assumed that adverse events lasted one day. Adverse effects of vancomycin, fidaxomicin and FMT via enema were assumed to be negligible and were not included in the model [32,33].

The analysis was performed from a societal perspective. The time horizon was 78 days. This duration was determined based on the duration of adverse events, the duration of initial therapy for the relapse, the expected time to relapse, and the duration of treatment for another relapse. This time horizon was chosen to put all the treatment strategies on an equal footing for the evaluation. At the end of the 78 days, patients could be in one of 5 health conditions: healthy, mild-to-moderate CDI, severe uncomplicated CDI, postcolectomy, and death. According to Van Nood et al.’s protocol, FMT treatment included a 4-day course of oral vancomycin [7], one day of transplant delivery and 2 days to resolution of symptoms. Dose and duration of all treatments are detailed in Table 1 and are consistent with published guidelines.

**Table 1:** Base case estimates, range, and distribution for model variables.

Variable	Base case value	Range	Distribution	Standard deviation	References
**Probabilities**					
Oral vancomycin pulse/taper—cure	0.771	0.652–0.890	Beta	0.061	[8,49]
Oral vancomycin pulse/taper—relapse	0.568	0.408–0.727	Beta	0.081	[8,49]
Fidaxomicin—cure	0.812	0.719–0.904	Beta	0.047	[21–25,50,51]
Fidaxomicin—relapse	0.211	0.105–0.316	Beta	0.054	[21–25,50,51]
FMT colonoscopy—cure	0.894	0.852–0.937	Beta	0.022	[8,52–58]
FMT colonoscopy—relapse	0.022	0.001–0.043	Beta	0.011	[8,52–58]
FMT colonoscopy after second FMT—cure	0.563	0.319–0.806	Beta	0.124	[8,52,55–57]
FMT duodenal infusion—cure	0.795	0.723–0.867	Beta	0.037	[7,26,59,60]
FMT duodenal infusion—relapse	0.021	0.000–0.049	Beta	0.014	[7,26,59,60]
FMT duodenal infusion after second FMT—cure	0.750	0.326–1.174	Beta	0.217	[7,26]
FMT enema—cure	0.833	0.712–0.955	Beta	0.062	[61–63]
FMT enema—relapse	0.000	0.000–0.000	Beta	0.000	[61–63]
FMT enema after second FMT—cure	0.500	0.100–0.900	Beta	0.204	[61,63]
Severe uncomplicated CDI	0.180	0.115–0.246	Beta	0.033	[64]
Severe complicated CDI	0.012	0.011–0.013	Beta	0.001	[36,64]
Colectomy	0.318	0.293–0.344	Beta	0.013	[36,37]
Postcolectomy mortality	0.407	0.350–0.463	Beta	0.029	[37,65–67]
Adverse events of FMT colonoscopy	0.002	0.000–0.012	Beta	0.005	[27–29]
Adverse events of FMT duodenal infusion	0.0005	0.000–0.002	Beta	0.0007	[31]
Mortality from FMT colonoscopy	0.0003	0.0002–0.0003	Beta	0.00003	[28–30,68]
Mortality from FMT duodenal infusion	0.0002	0.000–0.0004	Beta	0.0001	[31]
Hospitalization for mild CDI	0.000	0.000–0.000	Beta	0.000	Expert opinion
Mortality from mild CDI	0.007	0.002–0.012	Beta	0.002	[69,70]
Mortality from severe uncomplicated CDI	0.339	0.221–0.457	Beta	0.060	[71]
**Costs**^a^					
Oral vancomycin pulse/taper^b^	58	not varied			Local sources
Fidaxomicin (200 mg bid, 10 days)	1416	not varied			Local sources
Oral vancomycin (500 mg qid, 10 days)	50	not varied			Local sources
Intravenous metronidazole (500 mg tid, 10 days)	11	not varied			Local sources
Outpatient visit	43	not varied			Local sources
Donor and stool testing prior to FMT	825	not varied			NABM
Stool transplant preparation and traceability of samples	154	not varied			Local sources
Oral vancomycin (500 mg qid, 4 days) prior to FMT	20	not varied			Local sources
FMT delivery by colonoscopy	289	not varied			Local sources
FMT delivery by duodenal infusion	76	not varied			Local sources
FMT delivery by enema	5	not varied			Local sources
Follow-up outpatient visits	86	not varied			Local sources
Mean cost of hospitalization for mild-to-moderate CDI	2190	2099–2280	Gamma	45	[46]
Mean cost of hospitalization for severe CDI	8412	7725–9098	Gamma	343	[46]
Colectomy	719	not varied			CCAM
Adverse events of FMT colonoscopy	283	not varied			CCAM
Adverse events of FMT duodenal infusion	229	not varied			CCAM
**Utilities**					
Severe CDI (complicated or uncomplicated)	0.600	0.505–0.695	Beta	0.156	[47]
Mild-to-moderate CDI	0.782	0.628–0.936	Beta	0.154	[48]
Postcolectomy	0.536	0.382–0.690	Beta	0.154	[72]
Adverse events of FMT colonoscopy or FMT duodenal infusion	0.770	0.670–0.920	Beta	0.154	[73]
Healthy	1				
Death	0				

Abbreviations: bid: twice daily; CCAM: French Common Classification of Medical Procedures; CDI: *Clostridium difficile* infection; FMT: fecal microbiota transplantation; NABM: French Nomenclature of Procedures in Laboratory Medicine; IV: intravenous; od: once daily; qid: 4 times daily; tid: 3 times daily.

^a^Costs are reported as 2016 Euros.

^b^Oral vancomycin pulse/taper: oral vancomycin at 125 mg qid for 10 days, then 500 mg od every 2 days for 21 days.

### Model input parameters

Inputs for effectiveness data, costs, and utilities were pooled from published sources, which included clinical studies and systematic reviews. Additional input was sought from clinical experts for parameters for which data were limited, i.e. treatment pathway. Clinical experts were employees of the Lille University Hospital. All model variables are reported in Table 1.

#### Probabilities

We selected reports of treatments used to treat patients with multiple recurrent CDI (i.e. more than 2 episodes) from published sources. We used the protocol of pulsed-tapered vancomycin used in the only randomized controlled trial evaluating pulsed-tapered vancomycin published to date to define vancomycin cure and recurrence rates [8]. For treatment by fidaxomicin, reports were case series and retrospective cohort studies. We did not include randomized controlled trials evaluating the efficacy of fidaxomicin as patients with more than one occurrence of CDI within 3 months before the start of the trials were excluded from these studies [34,35]. For FMT, published studies including 2 randomized controlled trials were used [7,8]. We used cure rates of second-time FMT administration based on published studies. Because no data were available to define vancomycin and intravenous metronidazole cure, we used the probability of having a colectomy to define oral vancomycin plus intravenous metronidazole failure [36,37]. Incidence rates of severe CDI reported in the literature are often based on different definitions. We used the definition of severe CDI reported in European guidelines to define the probability of having severe uncomplicated CDI and severe complicated CDI [6]. Probability that a non-severe CDI was treated in hospital was assumed to be zero (infectious diseases physician input). Due to a paucity of data from published sources, it was assumed that treatments had the same probabilities of cure for mild and severe uncomplicated CDI.

#### Costs

All cost inputs are presented in 2016 Euro (€). Medication costs were obtained from prices negotiated by the largest central purchasing unit in the French public hospital sector. The cost of treatment with FMT included a single fecal transplantation procedure. Donor testing prior to FMT included routine laboratory screening, stool testing, and serologic testing. Donor testing was consistent with current guidelines (Table A in S1 File) [38]. The 2014 Annual report of the European Antimicrobial Resistance Surveillance Network (EARS-Net) was used to include rates of vancomycin-resistant Enterococci (VRE) and rates of carbapenem-resistant Enterobacteriaceae (CRE) in France [39].

Cost information for donor testing was obtained from the French Nomenclature of Procedures in Laboratory Medicine (NABM) [40]. The activity-based costing database for laboratories was used to value these costs. This database is reported by the French Directorate-General for Care Provision (DGOS) and provides a national benchmark of non-clinical cost data for hospitals, i.e. administrative, technical, and logistic data [41].

Costs of materials and technical time for stool transplant preparation and traceability of samples were provided by the department of Pharmacy of Lille University Hospital. Personnel costs were obtained using annual wage data reported by the French National Institute for Statistics and Economic Studies (INSEE) [42]. These costs were adjusted using the coefficient for calculating the total wage cost for each personnel category provided by the DGOS [43].

The cost of a colonoscopy procedure was provided by Lille University Hospital, according to their protocol. Cost of FMT via duodenal infusion included chest or abdominal radiograph to confirm correct placement of nasoduodenal tube. Cost of FMT via enema included the enema kit and the cost of the nurse performing the procedure. Cost of bowel preparation was included to all patients undergoing FMT, regardless of mode of delivery [44]. The cost of one outpatient visit was included in each treatment strategy. Cost of FMT included the procedure for donation. This included an outpatient visit for assessment of donor, an outpatient visit on the day of donation, a hospital letter to general practitioner, and a venipuncture for blood screening. FMT strategies also included two follow-up outpatient visits for recipients. Costs of visits for donors and recipients in FMT strategies were obtained from the French General Inspectorate of Social Affairs (IGAS) [45]. Hospital admission costs were obtained from the French National Costs Study (ENC) 2013 using the diagnostic related group “Severe digestive system diseases” [46].

#### Utilities

Given that utilities for patients with CDI are not currently available in the literature, we derived our quality-of-life estimate for CDI from utility of non-infectious diarrhea using European Quality of Life-5 Dimensions (EQ-5D) questionnaires [47,48]. Patients who were cured by pulsed-tapered vancomycin and fidaxomicin were assumed to spend 10 days in a state of mild-to-moderate or severe disease, and the subsequent duration of treatment in the healthy state, whereas patients who were cured by FMT were assumed to spend 7 days in disease state, i.e. the duration of treatment by FMT. Patients who were treatment failures remained in the initial disease state through the course of treatment.

### Analysis

Parameters were assumed to be independent. In addition to base case analysis, deterministic univariate sensitivity analyses were performed to investigate the impact of model uncertainties and robustness of our analysis. As FMT costs were based on standard costs in one hospital, ranges for these costs were not available. To account for uncertainty regarding these costs, a threshold analysis was performed to determine the variation in common costs of FMT that would change the strategies lying on the efficiency frontier.

In addition, because of the current debate regarding the superiority of the upper gastrointestinal route [74–76], we examined a scenario where cure and recurrence rates of FMT via duodenal infusion were the same as the ones of FMT via colonoscopy.

Finally, we conducted a probabilistic sensitivity analysis using 10,000 Monte Carlo simulations to simultaneously assess the effect of uncertainty in all parameters on model conclusions. Parameters used in sensitivity analyses appear in Table 1.

### Model outcome

The model outcome was the incremental cost-effectiveness ratio (ICER) expressed as cost per quality-adjusted life year (QALY) among the 5 strategies. No threshold is defined by French Health Authorities. Following the WHO’s Commission on Macroeconomics and Health [77], the GDP per capita (€32,000 in 2015 [78]) was used for interpreting the ICER. QALYs were obtained by multiplying the utility weight of a state by the time spent in that state. The study was designed, conducted, and reported in accordance with the Consolidated Health Economic Evaluation Reporting Standards (CHEERS) statement [79].

## Results

### Base case analysis

Base case analysis, using the mean value of each parameter, showed that FMT via enema was costlier and more effective than pulsed-tapered vancomycin, yielding an ICER of €18,092/QALY (Table 2). FMT via colonoscopy was costlier and more effective than FMT via enema, yielding an ICER of €73,653/QALY. Fidaxomicin and FMT via duodenal infusion were dominated by FMT via colonoscopy and FMT via enema: they were both more expensive and less effective than FMT via colonoscopy and FMT via enema.

**Table 2:** Base case analysis of competing strategies for the management of second recurrence of community-onset *Clostridium difficile* infection.

Treatment	Cost (€)	QALY	ICER
Vancomycin pulse/taper	1235	0.1812	
Fidaxomicin	2464	0.1988	(Dominated)
FMT via duodenal infusion	1834	0.2013	(Dominated)
FMT via enema	1610	0.2019	18,092^a^
FMT via colonoscopy	1816	0.2047	73,653^b^

Abbreviations: FMT: fecal microbiota transplantation; ICER: incremental cost-effectiveness ratio; QALY: quality-adjusted life year. Costs values are reported as 2016 Euros.

^a^ICER calculated for FMT via enema relative to pulsed-tapered vancomycin.

^b^ICER calculated for FMT via colonoscopy relative to FMT via enema.

### Sensitivity analyses

**Fig 2:**
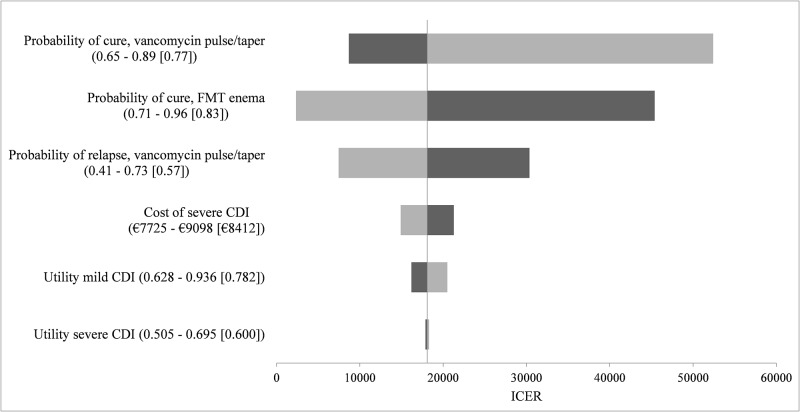
Tornado diagram, FMT via enema versus pulsed-tapered vancomycin. Name of the variable (lower bound of the parameter—higher bound of the parameter [base case]). The ICER corresponding to the lower parameter bound is shown in black, while the ICER corresponding to the higher parameter bound is shown in grey. This figure represents the impact of the uncertainty of six parameters on the base case results. Abbreviations: CDI: *Clostridium difficile* infection; FMT: fecal microbiota transplantation; ICER: incremental cost-effectiveness ratio.

Deterministic sensitivity analyses were used to explore the potential impact of six factors on the base case results: probabilities of cure and probability of relapse of vancomycin, probability of cure of FMT via enema, cost of severe CDI, and utilities of mild and severe CDI. Probability of cure and of relapse of pulsed-tapered vancomycin and probability of cure of FMT via enema, and to a lesser extent cost of severe CDI and utility of mild CDI, had an influence on the model (Fig 2). The model was sensitive to variation in probability of cure of FMT via enema. Indeed, varying this parameter within its stated range led to a change of sign in the ICER when considering FMT via enema versus FMT via colonoscopy: FMT via enema became either less effective and costlier that FMT via colonoscopy, or more effective and less costly than FMT via colonoscopy.

In addition, a threshold analysis was performed to find the variation in common costs of FMT that would change the strategies lying on the efficiency frontier. When common costs of FMT were increased by 81% of the baseline estimate, fidaxomicin (€2464, 0.199 QALY) was on the efficiency frontier, with an ICER of €69,890/QALY compared with pulsed-tapered vancomycin (€1235, 0.181 QALY), and FMT via enema (€2690, 0.202 QALY), compared with fidaxomicin, had an ICER of €72,212/QALY.

When considering a scenario where cure and recurrence rates of FMT via duodenal infusion were the same as the ones of FMT via colonoscopy, FMT via duodenal infusion (€1581, 0.2048 QALY) was on the efficiency frontier, with an ICER of €14,678/QALY compared with pulsed-tapered vancomycin, and both other routes of FMT administration were dominated.

**Fig 3:**
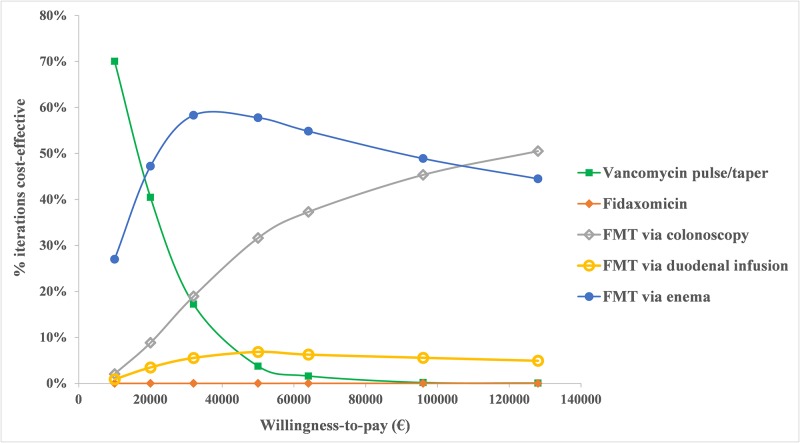
Acceptability curve of treatments of second recurrence of *Clostridium difficile* infection. This figure illustrates the proportion of the time each treatment was cost-effective at different willingness-to-pay thresholds. Abbreviations: FMT: fecal microbiota transplantation.

Probabilistic sensitivity analysis with 10,000 Monte Carlo simulations demonstrated that FMT via enema was the most cost-effective strategy in 58% of simulations and FMT via colonoscopy was favored in 19% at a willingness-to-pay threshold of €32,000/QALY. The cost-effectiveness acceptability curve displayed in Fig 3 shows the proportion of the time each treatment was cost-effective at various willingness-to-pay thresholds.

## Discussion

Our decision model indicated that the current standard approach using pulsed-tapered vancomycin is less costly than FMT, but FMT is more effective regardless of mode of delivery. The extra cost associated with FMT via enema for this increased effectiveness compared with vancomycin was €18,092/QALY. Thus, FMT via enema appears to be the most cost-effective strategy at a willingness-to-pay threshold of €32,000/QALY. The base case analysis showed that FMT via duodenal infusion and fidaxomicin were dominated by FMT via colonoscopy and FMT via enema. Fidaxomicin was on the efficiency frontier if common costs of FMT increased by 81%. However, such an increase in FMT costs is unlikely to occur.

Similar results have been demonstrated by other economic analyses evaluating FMT for treatment of recurrent CDI [10,14,15]. A cost-effectiveness analysis of strategies for treatment of recurrent CDI performed from a societal perspective in the USA concluded that FMT via colonoscopy was cost-effective with an ICER of $17,016 compared with vancomycin at a willingness-to-pay threshold of $50,000/QALY [10]. A recent study performed in Canada compared 6 strategies to treat recurrent CDI using the perspective of the Ontario Ministry of Health and Long-Term Care. The authors concluded that FMT via colonoscopy dominated all other strategies in the base case at a willingness-to-pay threshold of $50,000/QALY [15]. However, route of FMT often depends on the delivery method which is considered the safest for the patient [8]. Therefore, it appeared important to incorporate adverse events from colonoscopy and upper gastrointestinal endoscopy in our analysis. Our model also accounted for the utilities weights of these adverse events and for their respective associated costs. Another study by Varier et al. compared vancomycin with FMT for recurrent CDI from a third-party payer perspective using U.S. data. Even though the authors accounted for adverse events of colonoscopy, they found that FMT administered via colonoscopy was both less costly and more effective than prolonged oral vancomycin taper at all willingness-to-pay thresholds based on the probabilistic sensitivity analysis [14].

To our knowledge, this study is the first cost-effectiveness analysis investigating the use of treatment strategies including FMT for the treatment of second recurrence of community-onset CDI in France. Our analysis contains several limitations. First, we assumed that patients entering the model received outpatient treatment and we did not incorporate hospitalizations. However, it should be noted that patients with multiple comorbidities can be hospitalized for moderate CDI or can develop CDI while hospitalized, which was not captured by our model. Second, our conclusions are limited by the quality of the studies included. The lack of a standardized protocol for FMT administration leads to difficulties in comparison of efficacy across studies. For instance, we included studies which used less than 50 g of stool, although it is recommended that a large volume of stool be attempted [80]. This may have overestimated the recurrence rates of treatment with FMT. Third, we did not have a range for the majority of the costs included as these costs are not currently available at a national level and may also vary throughout countries. The Fidaxomicin/Vancomycin cost ratio is very high in our study compared to other cost-effectiveness studies, especially in those conducted in the USA [10,12]. Additionally, because only one study has stratified FMT results to *Clostridium difficile* ribotype 027 strain, we did not account for potential differences in treatment efficacy between NAP1/BI/027 and non-NAP1/BI/027 strains [56].

We could have added in our model a treatment by FMT as an alternative to colectomy for patients with severe complicated CDI, as it has been shown that FMT was also effective for these patients [81]. However, as this alternative is not mentioned in current guidelines, we did not consider it in our model. It should be noted that fidaxomicin could also be considered for treatment before FMT instead of vancomycin, although it would increase costs of FMT strategies. Indeed, fidaxomicin causes less disruption of the anaerobic microbiota than vancomycin, within the limitation of the method used to explore the intestinal microbiota [35,82]. Published studies used for this analysis were limited to immunocompetent patients. However, FMT has been shown to be safe, well tolerated, and effective also in immunocompromised patients [83,84]. Two recent randomized controlled trials have shown similar cure rates between frozen FMT and fresh FMT in treating recurrent CDI [74,85]. Using frozen FMT would reduce costs associated with donor screening frequency, provide immediate availability of the FMT, and enable delivery of the treatment to hospitals without on-site laboratory facilities [85].

In conclusion, this study, performed from a societal perspective, may give insights to healthcare decision makers when considering treatment for second recurrence of community-onset CDI.

## Supporting Information

S1 File Supplementary Table and Figure. (DOCX)
