# Supplementary material for: Cost-Effectiveness Analysis of Five Competing Strategies for the Management of Multiple Recurrent Community-Onset Clostridium difficile Infection in France
Source: PLoS One. 2017 Jan 19;12(1):e0170258. doi: 10.1371/journal.pone.0170258 (PMC5245822; doi:10.1371/journal.pone.0170258)
Supplement: S1 File — (DOCX) [file pone.0170258.s001.docx]

# Supporting Information

Table A. Donor laboratory testing prior to fecal microbiota transplantation.

| **Routine laboratory screening** | |
| --- | --- |
|  | Fasting glucose |
|  | Creatinine |
|  | Liver function tests (AST, ALT, GGT, ALP, bilirubin) |
|  | C-reactive protein |
|  | Complete blood count with differential |
|  | Liver function tests: coagulation (PT, PTT) |
| **Serologic testing** | |
|  | Syphilis serology |
|  | HIV 1/2 serology |
|  | HTLV serology |
|  | Hepatitis A, B, C, and E serology |
|  | CMV serology |
|  | *Strongyloides stercoralis* serology |
|  | Amebiasis serology |
|  | *Trichinella spiralis* serology |
| **Stool testing** | |
|  | *Clostridium difficile* toxin by PCR |
|  | Routine bacterial culture for enteric pathogens |
|  | Multidrug resistant (MDR) bacteria: vancomycin-resistant enterococci (VRE), carbapenemase-producing *Enterobacteriaceae* (CPE), extended-spectrum beta-lactamase (ESBL)-producing bacteria |
|  | Confirmation of resistance genes if MDR bacteria research is positive for VRE |
|  | Confirmation of resistance genes if MDR bacteria research is positive for CPE |
|  | Norovirus and rotavirus by multiplex RT-PCR |
|  | Parasites: *Cryptosporidium sp., Cyclospora sp., Giardia intestinalis, Isospora sp.* |
|  | *Strongyloides stercoralis* |
|  | Microsporidia |
|  | *Entamoeba histolytica* by PCR |

Abbreviations: ALP: alkaline phosphatase; ALT: alanine transaminase; AST: aspartate transaminase; CMV: cytomegalovirus; CPE: carbapenemase-producing Enterobacteriaceae; GGT: gamma-glutamyl transpeptidase; HIV: human immunodeficiency virus; HTLV: human T-lymphotropic virus; MDR: multidrug resistant; PCR: polymerase chain reaction; PT: prothrombin time; PTT: partial thromboplastin time; RT-PCR: reverse transcription-polymerase chain reaction; VRE: vancomycin-resistant enterococci.

Fig A-E. Decision tree comparing 5 strategies for the treatment of second recurrence of community-onset *Clostridium difficile* infection. Abbreviations: CDI: *Clostridium difficile* infection; IV: intravenous; FMT: fecal microbiota transplantation.

Fig A. Vancomycin pulse/taper arm. Abbreviations: CDI : *Clostridium difficile* infection; IV : intravenous.

Fig B. Fidaxomicin arm. Abbreviations: CDI : *Clostridium difficile* infection; IV : intravenous.

Fig C. FMT via colonoscopy arm. Abbreviations: CDI : *Clostridium difficile* infection; IV : intravenous; FMT: fecal microbiota transplantation.

Fig D. FMT via duodenal infusion arm. Abbreviations: CDI : *Clostridium difficile* infection; IV : intravenous; FMT: fecal microbiota transplantation.

Fig E. FMT via enema arm. Abbreviations: CDI: *Clostridium difficile* infection; IV: intravenous; FMT: fecal microbiota transplantation.
